# Sperm Chromatin Structure Assay (SCSA®) for Fertility Assessment

**DOI:** 10.1002/cpz1.508

**Published:** 2022-08-04

**Authors:** Donald P. Evenson

**Affiliations:** ^1^ SCSA Diagnostics Brookings South Dakota; ^2^ Department of OB/GYN, Sandford Medical School University of South Dakota Sioux Falls South Dakota

**Keywords:** DNA integrity, flow cytometry, male fertility, pregnancy outcomes, SCSA, sperm chromatin structure assay

## Abstract

The Sperm Chromatin Structure Assay (SCSA^®^) is a federally registered protocol for simultaneous flow cytometric measures of sperm DNA integrity and chromatin structure. Fresh or frozen/thawed raw semen samples are diluted in buffer to a sperm concentration of ∼1–2×10^6^ /ml and then treated with a pH 1.20 buffer for 30 s to open the DNA strands at sites of DNA strand breaks. The sperm are then stained with acridine orange (AO) that intercalates into double‐strand DNA and fluoresces green (515–530 BP filter) and stacks on single‐strand DNA that fluoresces red (630 LP filter) upon excitation from a 488 nm laser. The extent of single and double DNA strand breaks (DNA fragmentation index, %DFI) and level of excess nuclear histones (high DNA stainable sperm, %HDS) are simultaneously measured in individual sperm. From the time a fresh or frozen/thawed semen sample is received at the site of a flow cytometer (FCM) programmed for the SCSA protocol, data can be obtained within about 10 min on 5–10×10^3^ sperm. The %DFI and %HDS can be determined by computer‐gated regions on the green versus red cytogram. Alternatively, a determination is made by transforming the green versus red cytogram to a total DNA stainability (red + green fluorescence) versus red/red + green fluorescence cytogram from which a frequency histogram is produced and the %DFI calculated from it. The clinical threshold for human natural or IUI fertilization is 25% DFI at which point the ART lab should consider moving to ICSI fertilization. The clinical threshold for HDS is also 25%; values above this level may result in early embryo death due to abnormal gene readout caused by the abnormal tertiary structure of chromatin. Numerous lifestyle and environmental factors cause sperm DNA fragmentation. Reactive oxygen species (ROS) play a significant role in DNA breakage. © 2022 The Authors. Current Protocols published by Wiley Periodicals LLC.

**Basic Protocol 1**: Sperm Chromatin Structure Assay (SCSA®)

**Basic Protocol 2**: SCSA data analysis: Calculations of %DFI and %HDS of semen samples by one of two methods

**Support Protocol 1**: SCSA sample collection and shipping

**Support Protocol 2**: Flow cytometer set up

**Support Protocol 3**: Selection and use of reference samples

## INTRODUCTION

### Purpose of the SCSA test

The SCSA test is a two‐fold simultaneous flow cytometry measure of (1) the extent of sperm nuclear single (ss) and double (ds) strand DNA breaks and (2) chromatin structure, in thousands of sperm in a fresh or frozen/thawed semen sample. The extent of DNA strand breaks and chromatin structure abnormality is related to male factor fertility, including time to couple pregnancy, IVF embryo quality, miscarriage, or infertility.

### What problem(s)/knowledge gap does the SCSA test address?

Male fertility is classically addressed by semen tests that include sperm density, motility, and morphology. However, numerous studies over the past half‐century clearly show that, except for the absence of sperm, these parameters do not predict pregnancy since fertile and infertile men have overlapping values. The classical semen tests are light microscope measures of external sperm factors while the SCSA test measures internal nuclear factors not visible by light microscopy.

### What data can be obtained by executing the SCSA test?

- The extent of ss and ds DNA strand breaks
- The level of exchange of nuclear somatic histones for sperm‐specific protamines
- Relationship between the above factors and ART clinic‐derived and natural pregnancy outcomes
- Indicators of general male health
- Dose response to reproductive toxicants

### How do clinics/patients send semen samples to an SCSA diagnostic lab?

The SCSA test is a high precision test that requires a flow cytometer which, due to the need for a large capital expense and a trained technician, is not amenable to most infertility clinics. Thus, semen samples collected, as per detailed instructions, in a clinic or in a patient's home, can be flash‐frozen in a liquid nitrogen (LN_2_) dry shipper tank. (see Support Protocol 1). Clinics and/or patients go to a website such as www.scsatest.com (or other websites in other countries) and complete the order form. A pre‐cooled LN_2_ shipper is then sent to the clinic or home, the semen samples are flash‐frozen, and the LN_2_ shipper is then returned to an SCSA diagnostic testing lab via FedEx shipping. Some clinics send frozen semen samples in well‐insulated dry ice containers. (see Support Protocol 1). SCSA clinical data are then sent via a secure website to the physician or patient that ordered the test.

The SCSA test has only one protocol, described here in Basic Protocol 1. Two methods for analyzing the data are shown in Basic Protocol 2. Support Protocols are provided for preparation and shipping of samples, selection and use of reference samples, and instrument set‐up.

## Basic Protocol 1 SPERM CHROMATIN STRUCTURE ASSAY (SCSA®)

The SCSA test is a two‐fold simultaneous flow cytometric measure of (1) the extent of sperm nuclear single (ss) and double (ds) DNA breaks, and (2) chromatin structure, in thousands of sperm in a fresh or frozen/thawed semen sample.

### Materials

Fresh or frozen/thawed semen samples (see Support Protocol 1)
TNE buffer (see recipe)
Acid‐detergent solution (see recipe)
Acridine Orange (AO) stock solution, 1.0 mg/ml (see recipe)
AO staining buffer (see recipe)
AO staining solution (working solution) (see recipe)
AO equilibration buffer (see recipe)
FCM tubing cleanser (see recipe)
Purified water for reagents and FCM Sheath fluid (see recipe)

Water bath (37°C)
High‐resolution electronic balance
Adjustable, 0.20–0.80 ml automatic bottle dispenser for the acid‐detergent solution with glass amber bottle (Fisher Scientific)
Adjustable, 0.80–3.0 ml automatic bottle dispenser for the AO staining solution in glass amber bottle (Fisher Scientific)
Pipettors: adjustable 0–10 μl, 10–100 μl, 100–1000 μl, and a nonadjustable 200 μl (Fisher Scientific)
Vortex mixer
Tubes that fit the flow cytometer are used to mix the components of the SCSA test
Crushed ice buckets (3) for samples and reagent bottles
Container with ∼10% household bleach solution in which to place the unused portion of semen samples and used laboratory supplies
Stopwatch
Strongly suggested: a laminar flow biological safety hood to prepare the samples
35 L LN_2_ tank or ultracold (≥70°C) freezer
2‐ml cryotubes for freezing semen samples (Fisher)
0.5–1.0 ml snap cap cryotubes for storing numerous “reference samples” (Sarstedt)
Clinical specimen jars, polystyrene, sterile (VWR Scientific)
pH meter
Refrigerator (4°C) for reagents (*Do not use refrigerator freezer with automatic defroster to store semen samples because the rise and fall of temperature will cause DNA damage)*
LN_2_ dry shippers or well‐insulated dry ice containers to ship frozen semen samples
Fluorescent beads, Fluoresbrite, Plain YG 6 μ (Polysciences)
Aluminum foil
Disposable gloves (*human samples are handled using disposable gloves in a biological safety cabinet)*
Flow cytometer (FCM) set up as described in Support Protocol 2.
*The SCSA test requires only a minimally configured FCM consisting of a 488 nm laser and two detectors for green (FITC filter) and red (630LP filter) fluorescence and interfaced to a computer. Numerous makes and models are available worldwide*.
Ultracold freezer (−70° to −110°C) or, preferably, a 35 L LN_2_ tank

1 Fresh liquified semen samples can be diluted and measured immediately. Single frozen samples in cryotubes are immersed in a 37°C water bath, just until the last remnant of ice disappears.
2 Depending on sperm density in the semen samples, transfer a sufficient volume of fresh or flash‐frozen/thawed semen into 200 µl TNE buffer to attain an approximate 1–2×10^6^ sperm/ml.
  For many semen samples, a transfer of 5–10 µl is a good guess.
3 Add 400 μl acid detergent solution to the 200 μl sperm suspension. Mix on lab vortex.
4 After 30 s, add 1.20 ml AO staining solution with an automatic bottle dispenser. Mix on lab vortex.
5 Place the sample tube into the flow cytometer and start sample flow.
6 Start acquisition of FCM data at 3 min.
  This allows ample time for AO equilibration in the sample and hydrodynamic stabilization of the sample within the FCM fluidics, both important aspects of AO staining.
7 Check the sperm flow rate. If the rate is >300 cells/sec, a new sample is made at the appropriate dilution.
  This previous sample cannot be diluted with AO buffer to lower the concentration. A fresh sample must be prepared. Do not change the sample and sheath flow valve settings to increase or decrease the sample flow rate during an SCSA measurement period.
8 Collect 5000 or more FCM events. The actual number of sperm included in these measurements varies with the ratio of sperm to non‐sperm cells in the semen sample.
9 For verification of a proper SCSA measurement and for statistical considerations, samples should be independently measured with a second prepared sample in succession.
  It is important to measure one sample at a time rather than preparing several samples and placing the samples in an automatic carousel. Delayed measuring of prepared SCSA samples can produce artifacts for distinct reasons including ongoing oxidative stress causing further DNA breakage and absorbance of the AO stain to the sample tube altering the AO/DNA concentration required for differential staining of ss and ds DNA. Furthermore, measuring one sample at a time, followed by a second independent measure of an aliquot of the first sample, allows full control over each sample. If numerous samples were placed in an automatic carousel, any alteration of sample stream flow by a contaminating substance may ruin the entire batch. Since infertility clinics typically send only one frozen semen sample per patient, the thawed sample used hours ago may have deteriorated and no longer be of true value.
10 When measuring a batch of clinical samples, measure each sample as follows:
  - While the first measurement is being done, the repeat sample can be prepared and measured immediately after the first is completed.
  - Following the repeat sample, place the AO staining solution (see recipe) in the FCM and let that run until the next sample is ready. This step flushes out any adhering sperm in the fluidics and maintains equilibration of the AO with the fluidic sample tubing.
11 After the last sample has been measured, flush the FCM fluidic system with FCM tubing cleanser.

## Basic Protocol 2 SCSA DATA ANALYSIS: CALCULATIONS OF %DFI AND %HDS OF SEMEN SAMPLES BY ONE OF TWO METHODS

The flow cytometric SCSA test measures the percentage of sperm in a semen sample with broken (fragmented) DNA. This is termed the %DNA Fragmentation Index (%DFI). The SCSA test simultaneously measures the % of sperm in a semen sample with High DNA Stainability (%HDS). These sperm have an increased ratio of nuclear histones to protamines that causes an abnormal chromatin structure, which may cause abnormal readout of genes during early embryo growth.

Method 1 is done without a strict setup of the FCM for repeat measures with preset green and red fluorescence values. SCSA diagnostic labs that measure thousands of clinical semen samples use Method 2, in which all sample values are compared to a fixed reference sample set at fixed green and red fluorescence values on a linear 1024 × 1024 cytogram.

**Method 1**. For a quick measurement of one to a small series of samples without the use of a “reference sample” (see Support Protocol 3), place the AO‐stained semen sample in the flow cytometer, adjust the FCM green and red fluorescent gains so that the AO‐green fluorescent (515–530 nm BP) main population is just short of halfway up the Y axis on a linear 1024 × 1024 (or equivalent) cytogram and the AO‐red fluorescence (630 nm LP) is at about 1/5 of the total X axis.

**Figure 1:**
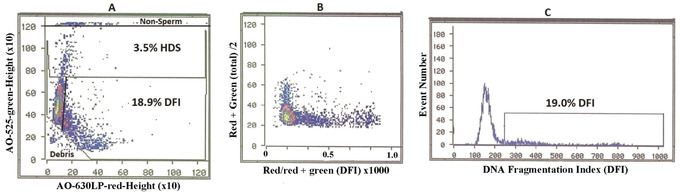
(**A**) AO**‐**Green (FITC 525 nm) versus AO‐Red (630 nm LP) fluorescence cytogram using an Ortho Cytofluorograf 30 FCM. Raw FCM data showing each of ∼5000 sperm as a single dot on scattergram. Y axis: green AO fluorescence of DNA stainability. X axis: red AO fluorescence of sperm with broken DNA. Dotted line at 75 on Y axis marks the upper boundary of DNA staining of normal sperm chromatin; above that line are sperm (dots) with partially uncondensed chromatin allowing more AO DNA stainability (HDS sperm). Computer gate along righthand portion of main sperm population without broken DNA. Computer gating shows 18.9% DFI and 3.5% HDS. Bottom left corner: gating out of apoptotic sperm with loss of DNA and seminal debris. Top horizontal line: gating out of non‐sperm somatic cells with diploid DNA content. (**B**) Raw data from left panel are converted by SCSAsoft (or equivalent software) to total AO DNA stainability (Y axis: red + green fluorescence/2) versus DNA fragmentation Index (X axis: red/red + green AO fluorescence × 1000). This transforms the angled sperm bullet display in the left panel to a vertical pattern that is used for sometimes more accurate determination of the percentage of sperm with fragmented DNA (%DFI) and transforms the DFI sperm to a horizontal pattern. **(C)** Frequency histogram of data from middle panel showing computer gating of %DFI. **Clinical report**: %DFI = 19.0 %HDS = 3.5.

Make four computer gates on the green versus red cytograms as seen in Figure 1A.

1. Make a horizontal gate near the top of the cytogram to exclude diploid somatic epithelial cells and leukocytes.
2. Make a horizontal gate touching the top of the cigar‐shaped main sperm population. Above this line are the sperm with an abnormal excess of histones (HDS fraction).
3. Make a straight or curved gate on the right side of the cigar‐shaped normal sperm population.
4. Make a 45‐degree angle gate that touches the bottom of the cigar‐shaped main population that excludes apoptotic and dead sperm and other seminal debris.
5. The region between 3 and 4 is the sperm with broken DNA (%DFI).
6. Use the computer to calculate %DFI and %HDS sperm.

**Method 2.** Conversion of green versus red cytogram (Fig. 1A) to a cytogram (Fig. 1B) of Y = green + red fluorescence (total)/2 versus X = red/red = green fluorescence (mean DFI = scale 0–1) × 1000.

From the Figure 1B cytogram, compute the frequency histogram (Fig. 1C) with the cut‐off point between sperm with and without broken DNA.

For human clinics, there is interest in only %DFI and %HDS. However, extensive use of other parameters (% moderate and high DFI, Mean DFI, and SD DFI) has been very useful in research studies of human and animal sperm as seen in many manuscripts (Evenson, 2016; Evenson, Baer, & Jost, 1989; Evenson & Wixon, 2005). Mean DFI (formerly termed “alpha t”) is calculated as red/red+ green fluorescence) and is a measure of the shift in FCM channels (0 to 1000) from green to red fluorescence (Darzynkiewicz, Traganos, Sharpless, & Melamed, 1975).

Our laboratory uses Method 2 as it lends itself to easy, rapid, and precise comparative determination of %DFI and %HDS on thousands of clinical samples via SCSAsoft or equivalent software. This method requires the use of a “reference sample” to set the FCM green and red fluorescent gains to near exact positions on the cytogram; for our Ortho FCM we set AO‐green at 475 ± 5/1024 and AO red at 125 ± 5/1024.

**Figure 2:**
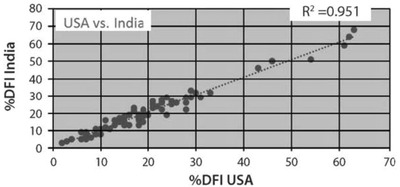
Correlations (R^2^ = 0.951) between %DFI measured from same frozen/ thawed human semen aliquots (n = 57) on an Ortho Cytofluorograf 30 FCM at SCSA Diagnostics in the USA and a FACSCalibur FCM at Andrology Center in Coimbatore, India. Reprinted with permission from Evenson (2018).

Figure 2 shows a high correlation (R^2^ = 0.951) between %DFI measures on human semen aliquots measured on two different FCMs in two different countries.

The SCSA test is a medical diagnostic test that must be certified by various national and state agencies, such as CLIA and the New York State Department of Health in the USA. Since our laboratory was the inventor of the SCSA test using the Ortho FCM, it was grandfathered as the test to be matched by others providing a clinical diagnosis. The flow cytometer brand must also be approved for SCSA testing. Thus, our laboratory will send to a new diagnostic center about 15–20 frozen semen aliquots that have been measured on our original Ortho FCM and these samples are then measured in the new diagnostic lab. The comparative data must match closely to that measured on our Ortho FCM or our certified FACScan FCM. Figure 2 shows the excellent correlations of %DFI on 57 semen samples obtained on two different FCMs.

## Support Protocol 1 SCSA SAMPLE COLLECTION AND SHIPPING

1 A physician, clinic, patient, or non‐patient can go to an SCSA Diagnostics testing website, for example, www.scsatest.com, or the websites of other SCSA diagnostic centers worldwide and click on: “order now”. Enter personal identifying details, name of clinic or physician if relevant, credit card number, and request to have a cooled LN_2_ dry shipper sent by FedEx to home or clinic delivery address and phone number.
  Prior to shipping a semen sample to an SCSA diagnostic center, it is necessary to do the following to ensure a “fresh” semen sample.
2 To obtain “fresh semen”, patients are advised to abstain for 2–3 days prior to collection. However, if the abstinence time was a week or two or more prior to this 2 to 3‐day abstinence period, then to obtain fresh sperm and not include dying/apoptotic sperm, we advise having two ejaculations, such as on Wednesday and Friday, and then collect a clinical sample on Monday. Alternatively, fresh sperm can be obtained by ejaculation morning and night for one day and then collect a clinical sample 2 days later. This is advised as sperm are stored in the epididymis after production in the testis, reactive oxygen species (ROS) activity, and the fact that the epididymis does not entirely empty all sperm upon ejaculation, meaning those retained sperm have increased DNA damage.
3 As per detailed instructions enclosed in the LN_2_ shipping tank, ejaculate by masturbation into a plastic clinical specimen container. Wait about 20–30 min for liquefaction of the semen. Freshly ejaculated semen consists of a large amount of a gel‐like substance within which the sperm are lodged. Normally this substance “liquefies” by semen proteases after approximately 30 min at room temperature and frees the sperm, thereby providing a homogenous suspension of sperm that ensures a random sampling of all sperm that then can be measured immediately by the SCSA test.
4a To ship frozen samples:
  Using the SCSA test kit enclosed plastic pipette, transfer about 0.25–0.35 ml semen into each of two or three 2.0‐ml cryovials. Tightly cap the cryovials and immediately flash freeze in the LN_2_ tank. Close the tank and seal with a zip tie. Contact FedEx (or equivalent service) for pickup of the LN_2_ tank to be delivered to a laboratory that performs SCSA testing (many in Asia, Europe, India, Scandinavia, and North and South America).
  Importantly, and in contrast to the WHO recommendation (WHO, 2021) raw clinical semen samples being sent for SCSA testing must be flash‐frozen **without dilution in buffer** or any cryoprotectants. (Note, however, that a semen sample that has been frozen with cryoprotectants yields the same data as those without cryoprotectants.)
  Semen samples may be kept for up to several hours at room temperature prior to measuring or freezing without significant loss of quality, allowing for collections within a medical institution and transport to the flow cytometry laboratory.
  Cryoprotectants are not needed since flash‐frozen sperm and those frozen with a cryoprotectant provide equivalent SCSA data. This feature is unique to mammalian sperm cells due to the highly condensed, crystalline nature of the nuclear chromatin.
4b To transport samples locally:
  If transport of freshly collected semen is required outside of a building complex to an FCM lab, the sample may be transported in an insulated box or jacket pocket to maintain ambient temperature, that is, to keep from freezing or put on liquid ice if the temperature is hot leading to increased %DFI not representative of fresh sperm.
  All human biological fluids are considered potentially infectious. Always observe precautions.
  i. Dry ice.

Semen samples shipped on dry ice are placed into a well‐insulated commercial shipping container. Small chunks of dry ice are first placed on the bottom of the shipping container, the sample box is placed near the center of the shipping box, and then more dry ice is placed around the box. Eight to 10 pounds (lbs) of dry ice, broken up into pieces, in an insulated shipping container are satisfactory for FedEx Priority overnight shipments from any point in the United States during any season. This amount of ice will keep samples frozen for at least 2–3 days; however, shipments should be made only on Monday through Wednesday in the rare event that the shipment is “mis‐shipped” or is held up for a day due to weather‐related problems. Dry ice shipments sent from London on Monday afternoon to our facility in South Dakota typically arrive on Wednesday.

Airline couriers have a hazardous goods restriction limiting the total amount of dry ice in a container to 5 lbs. By the time the box is picked up and transported to the airport, we estimate that the ice weight will be ∼5 lbs.

The advantage of shipping frozen semen samples in dry ice containers is that the shipping costs are only for one‐way shipping, thus reducing the cost of sample transport. The total weight of a dry ice shipper is less than the LN_2_ shipping tank, also leading to lesser shipping costs. The two disadvantages are that most clinics do not have dry ice easily available, and furthermore, the dry ice pack stays cold for only two to three days in contrast to LN_2_ shippers that stay cold for 7–10 days.

*ii*. LN_2_ shipper tanks.

These tanks can hold numerous 2.0‐ml cryotubes. The LN_2_ temperature holds for approximately 7–10 days depending on ambient temperatures. A label on the outside of the tank reads: DRY SHIPPER NON‐HAZARDOUS PER FX‐08 to meet airline regulations.

## Support Protocol 2 FLOW CYTOMETER SET UP

1 Ensure optical alignment of FCM using standard fluorescent beads (*see above)* as directed by instructions for the FCM used.
2 Very importantly, the AO equilibration buffer (see recipe) must be passed through the FCM fluidic system for ∼15 min to saturate the tubing with AO molecules that otherwise might be removed from the measured semen sample.
3 Using the reference semen sample (see Support Protocol 3), adjust the green and red fluorescent gains so that the mean green (AO – Green‐525 nm) and red (AO – red‐ 630 LP) fluorescence values are at 475/1024 ± 5 and 125/1024 ± 5 channels, respectively, or equivalent ratio in FCMs using a different scaling.
4 The green and red fluorescent signals are processed and displayed as peak (or height) rather than area signals.

## Support Protocol 3 SELECTION AND USE OF REFERENCE SAMPLES

To make strict comparisons between sperm samples from human fertility clinics (Evenson, Djira, Kasperson, & Christianson, 2020) or in animal or toxicology studies (Evenson, 2011, 2016, Evenson & Wixon, 2005), all samples need to have a reference point that requires precise repeat instrument settings whether done on measurements on the same or different days. These settings are obtained by using aliquots of a single semen sample called the “reference sample” (this is not a “control” sperm from a fertile donor). A semen sample that demonstrates heterogeneic DNA integrity (e.g., ∼15% DFI) is chosen as a reference sample and then diluted with cold (4°C) TNE buffer to a working concentration of 1–2×10^6^ cells/ml. Several hundred 300 μl aliquots of this dilution are quickly placed into 0.5‐ml snap‐cap vials and frozen at −70°C to −100°C in a freezer or, preferably, in an LN_2_ tank. These reference samples are used to set the red and green photomultiplier tube (PMT) voltage gains to yield the same mean red and green fluorescence levels from day to day. The mean red and green fluorescence values are set at ∼125/1024 ± 5 and ∼475/1024 ± 5 channels, respectively, or equivalent numbers depending on the scaling of the FCM. These settings allow for the DFI sperm to fall mostly at a downward 45° angle; this provides “room” on the cytogram to detect the increased levels of HDS and DFI of abnormal sperm.

The values established by a laboratory should be used consistently thereafter. Strict adherence to keeping the reference values in this range must be maintained throughout the measurement period. Obviously, it would be advantageous to prepare all batches of reference samples from the same individual donor. However, if a new donor is used, then first set the FCM PMTs from the previous reference sample to be at the same X and Y channel positions, then measure the new reference sample, note the red and green mean values, and use these values for the following studies. Since reference samples can be stored in LN_2_ for years, a donor could provide enough samples for thousands of reference aliquots.

## REAGENTS AND SOLUTIONS

Use appropriate chemical precautions when working with acridine orange, which is toxic. Use only the purest grade reagents and purified water (as below). All solutions and buffers are stored in a refrigerator at 4°C.

### Acridine Orange (AO) stock solution, 1.0 mg/ml

Acridine orange (AO) chromatically purified (e.g., Polysciences, Inc., Warrington, PA)
Dissolve AO powder in H_2_O at 1.0 mg/ml as follows:
Tare a 15 ml, flat‐bottom scintillation vial on a high‐resolution electronic balance.
Carefully remove and transfer 3–6 mg AO powder from the stock bottle with a micro spatula into the vial.
Add an exact equivalent number of milliliters of water.
Wrap the capped vial in aluminum foil to protect it from light.
Keep refrigerated for up to several months.
  AO is a toxic chemical and precautions must be taken when handling it.

### Acid‐detergent solution

20.0 ml 2.0 N HCl (0.08 N)
4.39 g NaCl (0.15 M)
0.5 ml Triton X‐100 (0.1%),
Add H_2_O taken up to a volume of 500 ml
Adjust pH to 1.20 with 5 N HCl.
Store at 4°C for up to several months.
  Use purchased 2.0 N HCl (e.g., Sigma); do not dilute from a more concentrated HCl solution that is likely less pure and may be of questionable strength.

### 0.10 M citric acid buffer

21.01 g citric acid monohydrate (F.W. = 210.14; 0.10 M)
Add H_2_O to a volume of 1.0 L
Store up to several months at 4°C.

### 0.20 M Na_2_PO_4_ buffer

28.4 g sodium phosphate dibasic (F.W. = 141.96; 0.20 M)
Add H_2_O to a volume of 1.0 L
Store up to several months at 4°C.
  Note that when the 0.20 M Na_2_PO_4_ buffer is removed from the refrigerator, salt crystals will be present. Heat in a 37°C water bath until the salts are fully dissolved.

### AO staining buffer

370 ml 0.10 M citric acid buffer
630 ml 0.20 M Na_2_PO_4_ buffer
372 mg EDTA (disodium, FW = 372.24; 1 mM)
8.77 g NaCl (0.15 M).
Mix overnight on a stir plate to ensure the EDTA is entirely in solution.
Adjust pH to 6.0 with saturated NaOH solution.
Store up to several months at 4°C.

### AO staining solution (working solution)

600 μl AO stock (1 mg/ml) solution is added to each 100 ml of staining buffer.
Keep this solution in the glass amber automatic dispenser for AO staining of samples.
Store up to 2–3 weeks at 4°C.

### AO equilibration buffer

400 μl acid‐detergent solution
1.20 ml AO staining solution.
  This solution is run through the FCM tubing for about 15 min prior to sample measurement to ensure that AO is equilibrated with the sample tubing.
  It is also run following the completion of a sample measurement and its duplicate, prior to measuring the next sample.

### TNE buffer

8.76 g NaCl (FW = 58.44; 0.15 M)
1.58 g Tris‐HCl (FW 158; 0.01 M)
0.37 g EDTA (disodium, FW 372.2; 1 mM)
Add H_2_O to 1.0 L
Adjust pH to 7.4 with 2.0 N NaOH
Store up to several months at 4°C

### FCM tubing cleanser

Used to unclog FCM sample lines and remove AO adhered to the FCM fluidics following use of multiuser FCM.
50:50 H_2_O: household bleach (contains ∼5% sodium hypochlorite)
0.5 M NaCl
Flush with sheath fluid.

### Purified water

Purified water is needed for all reagents and the FCM sheath fluid. It is highly recommended that city tap water is passed through a system like the Millipore system that contains cartridges of A. Super‐C carbon, B. Ion‐exchange, and Mill‐Pak (Millipore Sigma, Burlington, Mass). Depending on the purity of the city tap water, various treatments can be made prior to the Millipore system. Our laboratory passes tap water through a double water distiller before the Millipore. Other labs simply have a filter system prior to the Millipore. Other combinations are also available.

### Sheath fluid

Different FCM manufacturers recommend various agents to be added to the sheath fluid to keep the fluidic lines and flow cell clean. Our lab adds one drop of Finish® Jet‐Dry® to 4–6 L of sheath fluid (purified H_2_O) that aids in keeping the FCM fluidics clean. (*This product is a rinse aid liquid suspension of surfactants, salts, and acids used in home dishwashers. Found in home grocery stores*).

## COMMENTARY

### Background Information

#### Regulations for SCSA clinical testing

SCSA testing for clinical patients is strictly regulated by CLIA and other USA licensing agencies, such as New York State Department of Health. The lab director must have a High Complexity Laboratory Director (HCLD) certification. Numerous laboratory procedures and standards must be met and recorded. Flow cytometers must be certified to produce SCSA testing results. Per CLIA regulations, for every measurement period, a low %DFI and a high %DFI sample must be part of the measurement data. Equivalent regulations exist in other countries.

#### Implications and relevance of the SCSA test

Current data suggest that couple infertility is shared near 50:50 between the male and female partner (Agarwal et al., 2016). For the past many decades, the classical semen test has been the primary indicator of male factor fertility. Our introduction (Evenson, Darzynkiewicz, & Melamed, 1980) of the concept that sperm DNA fragmentation (SDF) and the SCSA test to measure SDF led the way to recognizing the significant role of sperm DNA and chromatin integrity in achieving successful couple pregnancy (Cho, Agarwal, Majzoub, & Esteves, 2017). Following the introduction of the SCSA test, three other SDF tests including the COMET, TUNEL, and SCD tests have been introduced and are now used worldwide (Esteves et al., 2021). All these tests measure the extent of DNA damage but only the SCSA test measures chromatin structure, which is thought to impact the three‐dimensional organization of chromatin, so when gene sequences needed to be read for proper embryo growth are not available, the embryo may die. While the other three SDF tests require up to twenty laboratory steps and hours of time, the SCSA test can be accomplished in minutes. Furthermore, the SCSA and TUNEL (FCM version) tests are measured by precise and objective flow cytometry, giving a higher statistical outcome and repeatability. While the requirement of a flow cytometer makes it a relatively high capital expense, the cost of reagents per SCSA test is measured in pennies while the other tests cost on the order of dollars for reagents and time/effort. Thus, researchers can run hundreds of SCSA tests for a few dollars and over short periods in contrast to the other tests.

#### Distinct features of the SCSA test

In sharp contrast to the protocols of the SDF tests that require up to twenty time‐consuming laboratory steps, the SCSA test requires only four quick simple steps accomplished in about 5 min: (1) Dilute fresh or frozen/thawed raw semen in buffer to ∼ 1–2×10^6^ sperm /ml, (2) treat with low pH buffer for 30 sec (3) stain with acridine orange (AO), (4) measure ∼5×10^3^ sperm by flow cytometry (FCM) to determine the extent of DNA strand breaks/sperm. The SCSA test has, for the past 30 years, had only one fixed, federally registered protocol: SCSA®. Also, in contrast to the light microscope measures of 50–500 sperm by the Comet, SCD, and TUNEL tests, the SCSA test measures 5000+ individual sperm by non‐biased FCMs, thus providing greater precision and statistical significance.

The SCSA test was the first pioneering sperm DNA fragmentation (SDF) test (Evenson, Darzynkiewicz, et al., 1980). Buffer‐diluted human or bull sperm nuclei in plastic test tubes were heated at 100°C in boiling water for 5 min. This opened (limited denaturation) the double‐strand DNA (dsDNA) at sites of single and double‐strand DNA breaks. This was followed by the addition of acridine orange (AO) that intercalates into native, double‐strand DNA and fluoresces green and stacks on ssDNA that fluoresces red upon exposure to 488 nm light as visualized by fluorescent light microcopy or measured in a flow cytometer (Evenson, Darzynkiewicz, et al., 1980).

Since the heat protocol of the original SCSA test was cumbersome and time‐consuming, the protocol presented here uses low pH to open up/denature the DNA at sites of single and double‐strand breaks, thus providing a protocol that can be accomplished within a few minutes and permits a high‐throughput assessment of clinical samples.

For the first years of using the pioneering SCSA test, the shift in the entire population from green to red fluorescence was monitored by the expression: mean alpha t (αt) = red/red + green fluorescence (Darzynkiewicz et al., 1975). However, since alpha t has a range of values from 0 to 1 (or sometimes scored ×10^3^), it is not a meaningful term for reports of sperm DNA integrity to human infertility clinics. Unfortunately, in the current 6^th^ edition of the WHO manual (WHO, 2021), this alpha t equation was incorrectly termed as %DFI. Therefore, the WHO manual provides no means or figures to calculate SCSA‐defined %DFI, that is, the % of sperm in the ejaculate with measurable DNA fragmentation. The calculation of %DFI as presented here is the correct means to report patient DNA integrity.

Numerous labs around the world utilize the SCSA test with diverse types of FCMs. By exchanging frozen semen samples between our lab and others, it has been shown that near exact repeats (Fig. 2) can be obtained between international labs using different flow cytometers (Evenson, 2016). These labs may process their raw SCSA data through SCSAsoft via a web link. Many manuscripts describe methods and outcomes of SCSA testing (Evenson, 2013, 2018; Evenson, Jost, Baer, Turner, & Schrader, 1991; Evenson, Larson, & Jost, 2002; Oleszczuk, Giwercman, & Bungum, 2016; Spano et al., 2000; Vaughan, Tirado, Garcia, Datta, & Sakkas, 2020).

The SCSA test protocol has been federally registered as the SCSA^®^ test and is known only as such in hundreds of manuscripts. In the current WHO manual, the SCSA^®^ test was misnamed the “acridine orange flow cytometry” test. While a few researchers have made minor modifications to the protocol as seen here, it is noted that precise concentrations of the various buffers and AO is critical to obtaining correct results. Thus, for any lab data to be compared with other worldwide SCSA data, the exact protocol shown here is strongly recommended.

### Critical Parameters/Troubleshooting

#### Cytogram patterns of SCSA data with different FCMs

The highly condensed mammalian sperm nucleus has a much higher index of refraction than sample sheath (water) in a flow cytometer. This differential, coupled with the typical non‐spherical shape of sperm nuclei and their orientation in the flow channel, produces an optical artifact consisting of an asymmetric, bimodal emission of DNA dye fluorescence when measured in orthogonal configuration flow cytometers; this is referred to as a “cigar‐shaped pattern” (Evenson, 2011). Since %DFI can be determined from a frequency histogram from data on a cytogram of red + green versus red/ red + green fluorescence as seen in Figure 1, the optical artifact of AO‐stained sperm measured in the orthogonal instruments does not interfere with the results and the %DFI frequency histogram is very narrow for a normal population of sperm. Although each type of flow cytometer with different configurations of lens and fluidics produces different cytogram patterns, the %DFI data are essentially the same (Evenson, 2016). %DFI and %HDS can also be determined by computer gating the regions on the cytogram as seen in Figure 1A.

In contrast to the WHO recommendations (WHO, 2021) that semen samples must be diluted with buffer before freezing and sent to a SCSA diagnostic center, it is strongly stated here from vast experience that the raw semen must be flash‐frozen in cryovials without dilution in buffer. This author recommends that the reader consider this Current Protocols article as the preferred reference for conducting the SCSA test for clinical patients.

### Understanding the Results

A review by Agarwal et al. (2016) outlined the evolution of sperm DNA fragmentation (SDF) tests from their origin to current utility in urology and infertility clinics and recognized that SDF has been generally acknowledged as a valuable tool for male fertility evaluation. These authors note that the American Urological Association (AUA) and the European Association of Urology (EWAU) have acknowledged the importance of DNA fragmentation in sperm as guidelines on male infertility. The authors conclude their review with the statement: “SDF testing should be included in the evaluation of male factor fertility along with the standard semen analysis. Any couple that fails to obtain a pregnancy within a year would gain a valuable insight into the potential that couple infertility may be due to sperm DNA fragmentation and, if so, to proceed with the recommendation to reduce SDF by lifestyle changes or select an ART procedure in part determined by the results of a SDF test.”

For *in vivo* pregnancies, a comprehensive clinical study of 165 presumably fertile couples (Evenson, Jost, Zinaman, et al., 1999), monitored over 12 menstrual cycles, were counseled on timely intercourse, and over the first 3 months all had an hCG test to monitor biochemical pregnancies. SCSA tests were done every month on all males until a pregnancy occurred for a total of 402 semen samples. SCSA alpha t values (now termed mean DFI) from 73 couples achieving pregnancy during months 1–3 of the study (group 1) were significantly different from those of 40 couples achieving pregnancy in months 4–12 (*p* < .01) and those of male partners of 31 couples not achieving pregnancy (*p* < .001). A total of 84% of males in group 1 had <15% DFI. This study and that of Spano et al. (2000) suggested an odds ratio of 8–10 for natural conception when the %DFI <30%.

**Figure 3:**
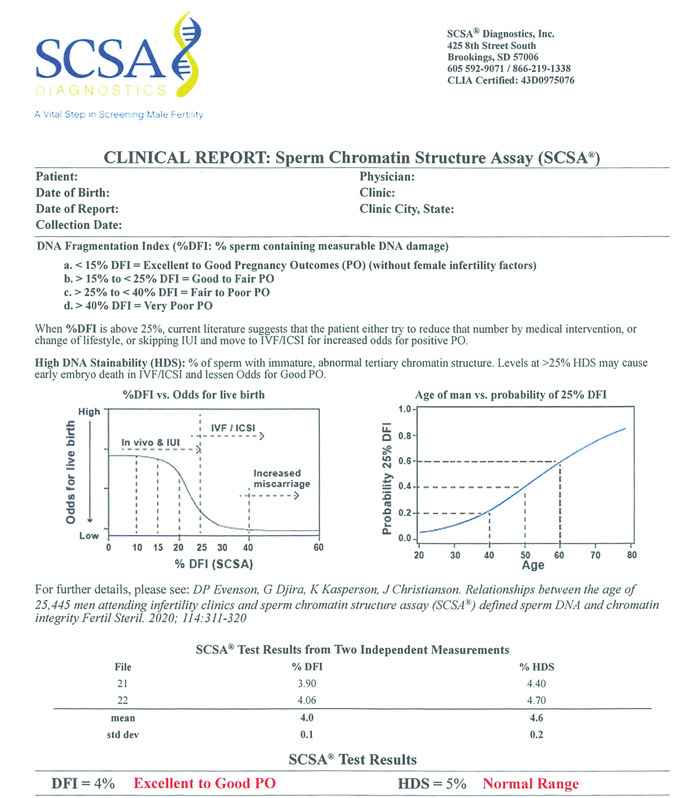
Example SCSA® Clinical Report. The clinical report identifies the patient by name, date of birth, date of semen collection, and date of SCSA measurement. Also shown is the name of the physician and clinic and its location. Different levels of %DFI are shown that correspond to the expected pregnancy outcomes (PO) without female infertility factors. Left figure: Estimated decreasing odds for producing a live birth via natural or IUI fertilization, moving from high odds at 0%–15% DFI to decreasing odds with >15% DFI. Right figure: Probability of reaching 25% DFI threshold levels at increasing ages. Two successive measures of all semen samples are made and the means and standard deviations of %DFI and %HDS are calculated. From those calculations the SCSA® Test Results are shown and, in red, the diagnosis is shown from the values above.

Other studies on natural and IUI fertilization showed thresholds for human male infertility risk at 25%–27% DFI (Bungum et al., 2007, Spano et al., 2000). As seen in Figure 3 (SCSA clinical report), if the male partner of the infertile couple has a %DFI of 0%–15%, the cause of the infertility is likely not due to abnormal SDF.

Bungum et al. (2007) observed that IUI success started to decrease at 20% DFI and approached zero when the DFI was >30%. Extensive studies by Erenpreiss, Elzanaty, and Giwercman (2008) and Oleszczuk et al. (2016) showed that the SCSA %DFI threshold for reduced fecundity appears to be at 20%. At 25% DFI, *in vivo* and IUI fertilization causes a dramatic fall in live birth rate. At this clinical threshold of ≥25%, the couple should be moved to ICSI fertilization. At even higher %DFI levels, the probability of miscarriage increases (Oleszczuk et al., 2016). Sperm DNA damage is clearly associated with an increased risk of pregnancy loss after IVF and ICSI (Zini, Boman, Belzile, & Ciampi, 2008). In the Evenson et al. (2020) study, 22.2% of the 25,445 men reached the clinical threshold of 25% DFI, and 10.6% reached the clinical threshold of 25% HDS. Whether this pattern can be changed by various therapeutic actions and individualized medicine is now a goal to achieve.

Infertility is a condition associated with multiple etiologies including not only SDF but also abnormalities of sperm nuclear chromatin structure. During human spermiogenesis, all but 15% of histones are replaced by protamines P1 and P2, resulting in sperm chromatin condensation followed by a halt to gene expression in haploid spermatids. Faulty compaction makes an abnormal tertiary chromatin structure that likely prevents the embryo from accessing the correct sequences of the paternal genome for proper initiation of the embryonic developmental program (Evenson, Jost, Corzett, & Balhorn, 2000).

High levels of sperm nuclear chromatin condensation abnormalities are associated with lower fertilization rates, impaired embryo quality, elevated arrested embryo rates, and decreased pregnancy rates. Sperm of men from repeated spontaneous abortion groups have been shown to have less chromatin condensation and poorer DNA integrity than sperm obtained from fertile men with no history of repeated spontaneous abortion (Jerre, Bungum, Evenson, & Giwercman, 2019).

The HDS factor as related to reduced pregnancy outcomes is much less known compared to the DFI factor. The level of green fluorescence as measured by the SCSA test is related to the condensation level of sperm chromatin and extent of restricted access of acridine orange staining. The sperm nuclear condensation process for mammals normally produces a 5‐fold reduction of DNA stainability relative to round spermatids. Lack of appropriate maturation results in HDS sperm with increased DNA stainability. When the %HDS is >25%, embryo growth *in vitro* and pregnancy outcomes are potentially compromised (Evenson, Djira, et al., 2020). Thus, HDS is an important factor to evaluate in the face of repeated ART failures and pregnancy outcomes.

**Figure 4:**
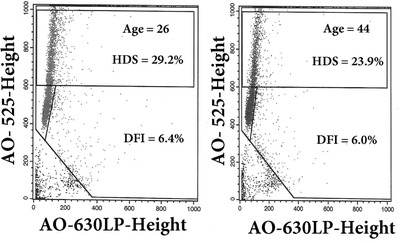
SCSA cytograms from FACScan FCM of semen cryopreserved at age 26 (left) and flash frozen raw semen at age 44 (right).

**Figure 5:**
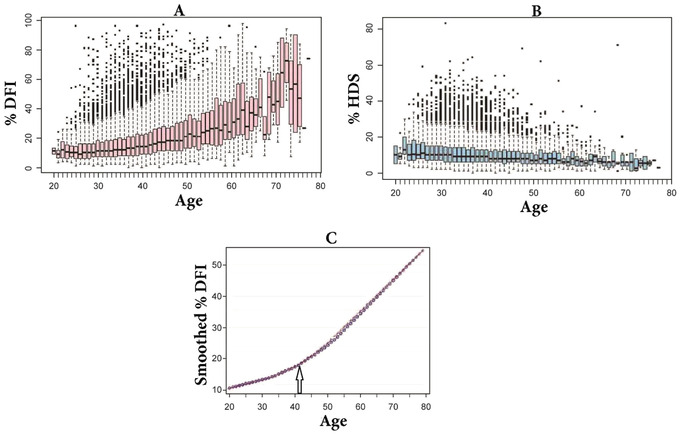
SCSA %DFI **(A)** and %HDS **(B)** data on flash frozen/thawed sperm from 25,445 men, ages 21–78, plotted as box plots. **(C)** %DFI as smoothed curve. Arrow at age 41 is point at which the rate of increase of %DFI doubles. (Reproduced from Evenson, Djira, et al., 2020 with permission.)

The HDS factor may be the primary alteration of sperm chromatin with normal %DFI. Figure 4 shows an example of a man who cryopreserved his sperm at age 26 and compared his SCSA test results at age 44. Both frozen/thawed samples were measured at the same time showing that there was no increase in %DFI (6.4% vs 6.0%) while there was a slight decrease in %HDS (29.2% vs 23.9%) consistent with the decrease of HDS with age (Fig. 5B). This figure shows that a man's SCSA profile can remain constant over a long period of time provided no exposure to agents known to alter DNA integrity and chromatin structure (Evenson, Jost, et al., 2000). Also of significant note, the sample frozen for nearly two decades had no deterioration in the frozen state. No attempts for a pregnancy were pursued during this time.

Further details on the HDS factor are found in a number of publications (Evenson, Djira, et al., 2020; Jerre et al., 2019; Menezo, Clement, & Amar, 2017).

#### Effects of age on SCSA‐measured parameters

Figure 5 shows the effects of age of 25,445 men on %DFI and %HDS (partial figure from Evenson, Djira, et al., 2020).

Data clearly show that a young age (e.g., 25–30) is no guarantee of high‐level DNA integrity. There is a great heterogeneity of %DFI and %HDS over the age span.

### Time Considerations

Nearly all aliquots of raw semen samples are flash frozen within 30–60 min after liquefaction. Thawed semen samples are immediately processed for SCSA testing.

Once the flow cytometer has been set up for running the SCSA test, a fresh or frozen/thawed semen sample can be run to acquire 5–10×10^3^ sperm in 5–10 min. The %DFI and %HDS can be determined immediately from computer gated regions on the cytogram or by processing through a computer program where a histogram of %DFI is determined as seen in Figure 1.

### Author Contributions

**Donald P Evenson**: Conceptualization, Formal analysis, Investigation, Methodology, Original draft writing, review, and editing.

### Conflict of Interest

The author is the President and Director of SCSA Diagnostics, Brookings, SD.

## Data Availability

The data that support the findings of this study are available on request from the corresponding author.

## References

[cpz1508-bib-0001] Agarwal, A. , Majzoub, A. , Esteves, S. C. , Ko, E. , Ramasamy, R. , & Zini, A. (2016). Clinical utility of sperm DNA fragmentation testing: Practice recommendations based on clinical scenarios. Translational Andrology and Urology, 5, 935–950. doi: 10.21037/tau.2016.10.03 28078226 PMC5182232

[cpz1508-bib-0002] Bungum, M. , Humaidan, P. , Axmon, A. , Spano, M. , Bungum, L. , Erenpreiss, J. , & Giwercman, A. (2007). Sperm DNA integrity assessment in prediction of assisted reproduction technology outcome. Human Reproduction (Oxford, England), 22, 174–179. doi: 10.1093/humrep/del326 16921163

[cpz1508-bib-0003] Cho, C. L. , Agarwal, A. , Majzoub, A. , & Esteves, S. C. (2017). The correct interpretation of sperm DNA fragmentation test. Translational Andrology and Urology, 6(Suppl 4), S621–S623. doi: 10.21037/tau.2017.06.25 29082980 PMC5643729

[cpz1508-bib-0004] Darzynkiewicz, Z. , Traganos, F. , Sharpless, T. , & Melamed, M. (1975). Thermal denaturation of DNA in situ as studied by acridine orange staining and automated cytofluorometry. Experimental Cell Research, 90, 411–428. doi: 10.1016/0014-4827(75)90331-6 46199

[cpz1508-bib-0005] Erenpreiss, J. , Elzanaty, S. , & Giwercman, A. (2008). Sperm DNA damage in men from infertile couples. Asian Journal of Andrology, 10, 786–790. doi: 10.1111/j.1745-7262.2008.00417.x 18645682

[cpz1508-bib-0006] Esteves, S. C. , Zini, A. , Coward, R. M. , Evenson, D. P. , Gosálvez, J. , Lewis, S. E. , … Humaidan, P. (2021). Sperm DNA fragmentation testing: Summary evidence and clinical practice recommendations. Andrologia, 53(2), e13874. doi: 10.1111/and.13874 33108829 PMC7988559

[cpz1508-bib-0007] Evenson, D. (2011). Sperm Chromatin Structure Assay (SCSA): 30 years’ experience with the SCSA. In A. Agarwal & A. Zini (Eds.), Sperm DNA and male infertility and ART. New York: Springer. doi: 10.1007/978-1-4419-6857-9_9

[cpz1508-bib-0008] Evenson, D. P. (2013). Sperm chromatin structure assay. In D. Carrell & K. Aston (Eds.), Spermatogenesis: Methods and protocols (Methods in Molecular Biology). New York: Humana Press.

[cpz1508-bib-0009] Evenson, D. P. (2016). The Sperm Chromatin Structure Assay (SCSA) and other sperm DNA fragmentation tests for evaluation of sperm nuclear DNA integrity as related to fertility. Animal Reproduction Science, 169, 56–75. doi: 10.1016/j.anireprosci.2016.01.017 26919909

[cpz1508-bib-0010] Evenson, D. P. (2018). Sperm Chromatin Structure Assay (SCSA®): Evolution from origin to clinical utility. In A. Zini & A. Agarwal (Eds.), A Clinician's Guide to Sperm DNA and Chromatin Damage. Springer.

[cpz1508-bib-0011] Evenson, D. P. , Baer, R. K. , & Jost, L. K. (1989). Long term effects of triethylenemelamine exposure on mouse testis cells and sperm chromatin structure assayed by flow cytometry. Environmental and Molecular Mutagenesis, 14, 79–89. doi: 10.1002/em.2850140203 2767059

[cpz1508-bib-0012] Evenson, D. P. , Darzynkiewicz, Z. , & Melamed, M. R. (1980). Relation of mammalian sperm chromatin heterogeneity to fertility. Science, 210, 1131–1133. doi: 10.1126/science.7444440 7444440

[cpz1508-bib-0013] Evenson, D. P. , Djira, G. , Kasperson, K. , & Christianson, J. (2020). Relationships between the age of 25,445 men attending infertility clinics and sperm chromatin structure assay (SCSA^®^) defined DNA and chromatin integrity. Fertility and Sterility, 114, 311–320. doi: 10.1016/j.fertnstert.2020.03.028 32653083

[cpz1508-bib-0014] Evenson, D. P. , Jost, L. K. , Baer, R. K. , Turner, T. W. , & Schrader, S. M. (1991). Individuality of DNA denaturation patterns in human sperm as measured by the sperm chromatin structure assay. Reproductive Toxicology, 5, 115–125. doi: 10.1016/0890-6238(91)90039-I 1807542

[cpz1508-bib-0015] Evenson, D. P. , Jost, L. K. , Corzett, M. , & Balhorn, R. (2000). Characteristics of human sperm chromatin structure following an episode of influenza and high fever: A case study. Journal of Andrology, 21, 739–746. 10975421

[cpz1508-bib-0016] Evenson, D. P. , Jost, L. K. , Zinaman, M. J. , Clegg, E. , Purvis, K. , de Angelis, P. , & Clausen, O. P. (1999). Utility of the Sperm Chromatin Structure Assay (SCSA) as a diagnostic and prognostic tool in the human fertility clinic. Human Reproduction, 14, 1039–1049. doi: 10.1093/humrep/14.4.1039 10221239

[cpz1508-bib-0017] Evenson, D. P. , Larson, K. , & Jost, L. K. (2002). The Sperm Chromatin Structure Assay (SCSA): Clinical use for detecting sperm DNA fragmentation related to male infertility and comparisons with other techniques. Journal of Andrology, 23, 25–43. doi: 10.1002/j.1939-4640.2002.tb02599.x 11780920

[cpz1508-bib-0018] Evenson, D. P. , & Wixon, R. (2005). Environmental toxicants cause sperm DNA fragmentation as detected by the Sperm Chromatin Structure Assay (SCSA). Toxicology and Applied Pharmacology, 207, S532–S537. doi: 10.1016/j.taap.2005.03.021 15987647

[cpz1508-bib-0019] Jerre, E. , Bungum, M. , Evenson, D. , & Giwercman, A. (2019). Sperm chromatin structure assay high DNA stainability sperm as a marker of early miscarriage after intracytoplasmic sperm injection. Fertility and Sterility, 12, 46–53. doi: 10.1016/j.fertnstert.2019.03.013 31043234

[cpz1508-bib-0020] Menezo, Y. , Clement, P. , & Amar, E. (2017). Evaluation of sperm DNA structure, fragmentation and decondensation: An essential tool in the assessment of male infertility. Translational Andrology and Urology, 6, S553–6. doi: 10.21037/tau.2017.03.11 29082177 PMC5643722

[cpz1508-bib-0021] Oleszczuk, A. , Giwercman, A. , & Bungum, M. (2016). Sperm chromatin structure assay in prediction of in vitro fertilization outcome. Andrology, 4, 290–296. doi: 10.1111/andr.12153 26757265

[cpz1508-bib-0022] Spano, M. , Bonde, J. P. , Hjollund, H. I. , Kolstad, H. A. , Cordelli, E. , & Leter, G. (2000). Sperm chromatin damage impairs human fertility. The Danish first pregnancy planner study team. Fertility and Sterility, 73, 43–50. 10632410 10.1016/s0015-0282(99)00462-8

[cpz1508-bib-0023] Vaughan, D. A. , Tirado, E. , Garcia, D. , Datta, V. , & Sakkas, D. (2020). DNA fragmentation of sperm: A radical examination of the contribution of oxidative stress and age in 16,945 semen samples. Human Reproduction, 35, 2188–2196. doi: 10.1093/humrep/deaa159 32976601

[cpz1508-bib-0024] WHO . (2021). 6th edition of WHO laboratory manual for the examination and processing of human semen. Retrieved from https://www.who.int/publications/i/item/9789240030787

[cpz1508-bib-0025] Zini, A. , Boman, J. M. , Belzile, E. , & Ciampi, A. (2008). Sperm DNA damage is associated with an increased risk of pregnancy loss after IVF and ICSI: Systematic review and meta‐analysis. Human Reproduction, 23, 2663–2668. doi: 10.1093/humrep/den321 18757447

